# LMI-Based Delayed Output Feedback Controller Design for a Class of Fractional-Order Neutral-Type Delay Systems Using Guaranteed Cost Control Approach

**DOI:** 10.3390/e24101496

**Published:** 2022-10-19

**Authors:** Zahra Sadat Aghayan, Alireza Alfi, António M. Lopes

**Affiliations:** 1 Faculty of Electrical Engineering, Shahrood University of Technology, Shahrood 36199-95161, Iran; 2 LAETA/INEGI, Faculty of Engineering, University of Porto, 4200-465 Porto, Portugal

**Keywords:** fractional calculus, stability, neutral system, delay, guaranteed cost control

## Abstract

In this research work, we deal with the stabilization of uncertain fractional-order neutral systems with delayed input. To tackle this problem, the guaranteed cost control method is considered. The purpose is to design a proportional–differential output feedback controller to obtain a satisfactory performance. The stability of the overall system is described in terms of matrix inequalities, and the corresponding analysis is performed in the perspective of Lyapunov’s theory. Two application examples verify the analytic findings.

## 1. Introduction

Delay is a pervasive issue that affects substantially the performance of the dynamical systems. Thus, the stability analysis of delayed systems has gained a renewed interest [1,2,3]. Neutral-type systems are general versions of delayed systems, where the delay occurs in the system states and their derivatives simultaneously [4]. A number of physical phenomena are modeled by neutral delay differential equations, such as population ecology [5], circuits [6] and network-based control systems [7].

Fractional calculus extends the integrodifferential calculus to noninteger orders [8] and was revealed to be powerful in the modeling and control of real-world problems [9,10,11,12,13]. Indeed, the tools of fractional calculus can model the dynamical behavior of many systems more exactly than the ones provided by integer-order calculus, and we find a number of applications in areas such as biology [14], energy storage [15], physics [16], signal and image processing [17,18], mechanical systems [19], and heat flow in a porous media [20]. Therefore, the stability analysis of fractional-order (FO) systems is an interesting problem [21,22,23,24,25,26].

FO neutral-type delay systems are more general than other types of delayed systems [27]. Thus, the stabilization of such systems for both integer- [28,29] and fractional-order [30,31,32,33,34] systems is a challenging topic. In the real world, mechanical and electrical components can cause a time delay in actuators. When changing the input command, the delay effect is apparent in the system behavior. Accordingly, the input delay should be considered in the design of the control system.

Ignoring uncertainties in the dynamical models, such as parameter perturbation, can significantly compromise the controller design procedure [35]. Thus, the effects of this on the system’s stability and dynamical behavior have been examined in the literature [36]. Accordingly, a robust control algorithm is needed, while guaranteeing the system’s stability and sufficient performance. To address this issue, a good technique is the guaranteed cost control (GCC) [37], which constructs an upper bound on a predefined index. A linear matrix inequality (LMI) is an effective manner to address the GCC of dynamical systems [38,39,40,41,42,43,44]. The GCC technique was adopted to stabilize teleoperation systems with uncertainty in [39]. The GCC-based synchronization of complex networks was addressed in [40]. The GCC of neutral-type dynamical systems with uncertainty was investigated in [38]. The GCC problem of a class of FO delayed systems using a state-feedback controller was reported in [45]. The stabilization of singular systems with delay using the GCC was addressed in [46]. The GCC problem of FO neural networks was addressed in [43,44]. The stability of certain and uncertain nonlinear systems with delay using GCC was discussed in [41,42]. The GCC of cellular neural networks with different properties was investigated in [47,48]. A GCC-based feedback control system for uncertain neutral systems was designed in [49]. Nonetheless, we observe that most of the works [46,47,48,49,50] focus on integer-order systems. Moreover, the feedback controller design of uncertain FO neutral-type delay systems via GCC has rarely been discussed.

A great deal of works reporting on the stability of neutral systems adopted a state-feedback controller [31,32,33,34,51,52]. However, it is possible that not all the system’s states are accessible. This issue points to the use of the output feedback control technique [53]. Simplicity with regard to the problem formulation is the key reason to choose a static output feedback. In addition, some classes of dynamic compensators can be described as a static output feedback. We can mention, for example, the proportional–integral–derivative (PID) controller. This controller has useful properties, such as the simplicity of its architecture and a low computational complexity. These characteristics make it easier to use PID instead of advanced controllers and, therefore, they are broadly applied in different engineering problems [54]. In particular, we can achieve a global asymptotic stabilization of the system using a PD controller. This is due to its conceptual simplicity and explicit tuning rules [55,56]. Table 1 and Table 2 summarize the key differences with respect to FO model characteristics and the designed controller.

In the present work, we study the GCC of uncertain FO neutral systems with delayed input via an output feedback PD control design. The closed-loop system stability conditions are formulated in terms of LMIs via the Lyapunov stability concept. To the best of our knowledge, this problem has not yet been explored in the literature. The proposed control approach is applied in two test cases and its performance is verified.

This paper has seven Sections. In Section 2 and Section 3, some preliminary concepts and the problem under study are introduced, respectively. In Section 4, the controlled system stability is analyzed. In Section 5 and Section 6, the applicability of the proposed control strategy is verified. In Section 7, the conclusions are outlined.

In the following, a diagonal matrix and an identity matrix with appropriate dimension are represented by $diag\left\{\cdot \right\}$ and *I*, respectively, and ∗ represents the symmetric component of any matrix.

## 2. Prerequisites

**Definition** **1** ([57])**.** *A derivative of a continuous function ϑ(t) is given by*

(1) $${}^{C}D_{}^{q}\vartheta \left(t\right)=\frac{1}{\Gamma (p-q)}\int_{t_{0}}^{t}{\left(t-\theta \right)}^{p-q-1}\vartheta^{\left(p\right)}\left(\theta \right)d\theta ,$$

*where q∈R stands for the fractional order, Γ(q) is the Gamma function, and $p\in Z^{+}$ satisfies 0≤p−1≤q<p.*

**Definition** **2** ([38])**.** Let us consider a cost function J. Given the existence of a control law $u^{⋆}\left(t\right)$ and a positive scalar $J^{⋆}$ for all permissible uncertainties and a specified delay, the overall asymptotical stability is guaranteed. Moreover, if $J<J^{⋆}$ holds, $u^{⋆}\left(t\right)$ and $J^{*}$ are named the GCC law and GCC value for the system, respectively.

**Lemma** **1** ([58])**.** *For the matrices $\Lambda_{1},\Lambda_{2}$, and $\Lambda_{3}$, where $\Lambda_{1}={\Lambda_{1}}^{T}$ and $\Lambda_{2}>0$, we have $\Lambda_{1}+{\Lambda_{3}}^{T}{\Lambda_{2}}^{-1}\Lambda_{3}<0$ if and only if*

(2) $$\left(\begin{array}{cc} \Lambda_{1} & {\Lambda_{3}}^{T}\\ \Lambda_{3} & -\Lambda_{2} \end{array}\right)<0\;\;\mathrm{or}\;\;\left(\begin{array}{cc} {-\Lambda }_{2} & \Lambda_{3}\\ {\Lambda_{3}}^{T} & \Lambda_{1} \end{array}\right)<0.$$

**Lemma** **2** ([59])**.** *For real matrices with appropriate dimensions H,N, and F and any matrix M(t) with $\;M^{T}\left(t\right)M\left(t\right)\leq I$, there exists*

(3) $$F^{T}M^{T}\left(t\right)H^{T}+HM\left(t\right)F+N<0,$$

*on the existence condition of a positive scalar γ satisfying*

(4) $$\gamma HH^{T}+\gamma^{-1}F^{T}F+N<0.$$

**Lemma** **3** ([60])**.** *For a given differentiable vector-valued function $\vartheta \left(t\right)\in R^{n}$ and $q\in \left(0,1\right)$, the following relationship holds*

(5) $${}^{C}D_{}^{q}\left(\vartheta^{T}\left(t\right)G\vartheta \left(t\right)\right)\leq {\left(\vartheta^{T}\left(t\right)G\right)}^{C}D_{}^{q}\vartheta \left(t\right)\;+{\left({}^{C}D_{}^{q}\vartheta \left(t\right)\right)}^{T}G\vartheta (t),$$

*where $G\in R^{n\times n}$ is a symmetric positive-definite matrix.*

**Lemma** **4** ([61])**.** *Suppose that for a given delayed system*

(6) $${}^{C}D^{q}G\left(t\right)=f\left(t,G_{t}\right),$$

*with $G_{t}=G\left(t+\theta \right)$ and −δ≤θ≤0, the functions $\left\{\xi_{1},\xi_{2},\xi_{3}\right\}:R^{+}\to R^{+}$ are nondecreasing continuous. The functions $\xi_{1}\left(g\right)$ and $\xi_{2}\left(g\right)$ are also positive for g>0, in which $\xi_{1}\left(0\right)=\xi_{2}\left(0\right)=0$ and $\xi_{2}$ is strictly increasing. Given the existence of a continuously differentiable function $V:R^{+}\times R^{n}\to R^{+}$ and a constant α>1 so that*

(7) $$\begin{array}{cc} \; & \left(1\right)\;\;\xi_{1}\left(∥G∥\right)\leq V\left(t,G\right)\leq \xi_{2}\left(∥G∥\right),\\ \; & \left(2\right)\;{\;}^{C}D^{q}V\left(t,G\left(t\right)\right)\leq -\xi_{3}\left(∥G\left(t\right)∥\right)\;\;if\;\;V\left(t+\delta ,G\left(t+\delta \right)\right)\leq \alpha V\left(t,G\left(t\right)\right),\;t\geq 0,\;\forall \delta \in \left[-\varrho ,0\right], \end{array}$$

*the zero solution of (6) is asymptotically stable.*

**Lemma** **5** ([62])**.** *For the given vectors β and $\alpha \in R^{n}$, we have*

(8) $$\pm 2\alpha^{T}\beta \leq \alpha^{T}\epsilon \alpha +\beta^{T}\epsilon^{-1}\beta .$$

*where ϵ is any real positive-definite matrix.*

## 3. Problem Statement

We study the uncertain FO neutral-type delay systems described in state-space by

(9) $$\begin{array}{c} \begin{array}{cc} \; & {}^{C}D_{}^{q}\vartheta \left(t\right)=\left(A_{0}+\Delta A_{0}\left(t\right)\right){\;}^{C}D_{}^{q}\vartheta \left(t-\delta \right)+\left(A+\Delta A\left(t\right)\right)\vartheta \left(t\right)+\\ & \left(A_{d}+\Delta A_{d}\left(t\right)\right)\vartheta \left(t-\delta \right)+\left(B+\Delta B\left(t\right)\right)u\left(t\right),\;q\in \left(0,1\right),\\ & y(t)=C\vartheta (t),\\ & \vartheta (t)=\phi (t),\;t\in [-\delta ,0], \end{array} \end{array}$$

in which $y\;\;\left(t\right)\in R{\;}^{v}$, $u\;\left(t\right)\in R^{m}$, and $\vartheta \left(t\right)\in R^{n}$, respectively, denote the output, the input, and the state vectors, δ represents the constant delay, and $A,A_{d},A_{0},B$, and C are known real matrices with compatible dimensions; the uncertainty terms are given by

(10) $$\begin{array}{c} \begin{array}{cccc} \left[\Delta A(t\right) & \Delta A_{d}\left(t\right) & \Delta A_{0}\left(t\right) & \Delta B(t) \end{array}]=HM\left(t\right)[\begin{array}{cccc} E_{0} & E_{1} & E_{2} & E_{3}], \end{array} \end{array}$$

where $H,E_{0},E_{1},E_{2}$, and $E_{3}$ are known real matrices, and M(t) denotes the time-varying parametric uncertainties satisfying $M^{T}\left(t\right)M\left(t\right)\leq I$.

Here, the guaranteed cost output feedback PD design for (9) is studied, to guarantee its asymptotic stability, using the following objective function

(11) $$\begin{array}{c} J=\frac{1}{\Gamma (q)}\int_{0}^{h}{\left(h-\sigma \right)}^{q-1}(\vartheta^{T}\left(\sigma \right)Q_{1}\vartheta \left(\sigma \right)+u^{T}\left(\sigma \right)Q_{2}u\left(\sigma \right))d\sigma ,\;h>0, \end{array}$$

where $Q_{1}$ and $Q_{2}$ are positive-definite symmetric matrices.

## 4. Theoretical Results

Hereafter, we adopt a delayed output feedback PD controller as

(12) $$\begin{array}{c} u\left(t\right)=K_{p}y\left(t-\delta \right)+K_{d}\;{}^{C}D_{}^{q}y\left(t-\delta \right), \end{array}$$

where $K_{d}$ and $K_{p}$ denote, respectively, the derivative and proportional output feedback gain matrices.

Then, we can express (12) as

(13) $$\begin{array}{c} u\left(t\right)=K_{p}C\vartheta \left(t-\delta \right)+K_{d}C\;{}^{C}D_{}^{q}\vartheta \left(t-\delta \right). \end{array}$$

The overall system (9), using the controller in (13), is then expressed by

(14) $$\begin{array}{c} \begin{array}{cc} \; & {}^{C}D_{}^{q}\vartheta \left(t\right)=\left(A+\Delta A\right)\vartheta \left(t\right)+\left(A_{d}+\Delta A_{d}+BK_{p}C+\Delta BK_{p}C\right)\vartheta \left(t-\delta \right)\\ & \;\;\;\;\;+{\left(A_{0}+\Delta A_{0}+BK_{d}C+\Delta BK_{d}C\right)}^{C}D_{}^{q}\vartheta \left(t-\delta \right),\\ \; & \vartheta (t)=\phi (t),\\ \; & t\in [-\delta ,0]. \end{array} \end{array}$$

Next, we determine the gains of the output feedback PD controller, i.e., $K_{p}$ and $K_{d}$, such that the entire system (14) is robustly asymptotically stable, where the minimum upper bound of J given in (11) is guaranteed.

Theorem 1 provides the asymptotic stability criteria of (14), which are described with the help of matrix inequality.

**Theorem** **1.** *Consider dynamical system (14) with matrices $Q_{i}$ (i=1,2) in (11), which are symmetric positive-definite. Given the existence of any appropriately dimensioned matrices X,Y, a positive scalar λ, a symmetric positive-definite matrix $\tilde{P}$, and a diagonal positive matrix Σ with*

(15) $$\begin{array}{c} \left(\begin{array}{ccccccccccc} \Theta_{11} & \Theta_{12} & \Theta_{13} & \tilde{P}A^{T} & \tilde{P}{E_{0}}^{T} & \lambda H & \tilde{P}Q_{1} & 0 & 0 & 0 & 0\\ {}* & -\tilde{P} & \Theta_{23} & \Theta_{24} & \Theta_{25} & 0 & 0 & X^{T} & X^{T} & 0 & 0\\ {}* & * & \Theta_{33} & \Theta_{34} & \Theta_{35} & -\lambda H & 0 & 0 & 0 & Y^{T} & Y^{T}{Q_{2}}^{T}\\ {}* & * & * & -2\tilde{P} & 0 & \lambda H & 0 & 0 & 0 & 0 & 0\\ {}* & * & * & * & -\lambda I & 0 & 0 & 0 & 0 & 0 & 0\\ {}* & * & * & * & * & -\lambda I & 0 & 0 & 0 & 0 & 0\\ {}* & * & * & * & * & * & -Q_{1} & 0 & 0 & 0 & 0\\ {}* & * & * & * & * & * & * & -Q_{2}^{-1} & 0 & 0 & 0\\ {}* & * & * & * & * & * & * & * & -\Sigma^{-1} & 0 & 0\\ {}* & * & * & * & * & * & * & * & * & -Q_{2}^{-1} & 0\\ {}* & * & * & * & * & * & * & * & * & * & -\Sigma \end{array}\right)<0, \end{array}$$

*where*

$$\begin{array}{c} \begin{array}{cc} \; & \Theta_{11}=A\tilde{P}+\tilde{P}A^{T}+\tilde{P},\;\;\Theta_{12}=A_{d}\tilde{P}+BX,\;\;\Theta_{13}=A_{0}\tilde{P}+BY-\tilde{P}A^{T},\\ \; & \Theta_{23}=-\tilde{P}{A_{d}}^{T}-X^{T}B^{T},\;\;\Theta_{24}=\tilde{P}{A_{d}}^{T}+X^{T}B^{T},\;\;\Theta_{25}=\tilde{P}{E_{1}}^{T}+X^{T}{E_{3}}^{T},\\ \; & \Theta_{33}=-2\left(A_{0}\tilde{P}+BY\right),\;\;\Theta_{34}=\tilde{P}+\tilde{P}{A_{0}}^{T}+Y^{T}B^{T},\;\;\Theta_{35}=\tilde{P}{E_{2}}^{T}+Y^{T}{E_{3}}^{T}, \end{array} \end{array}$$

*then the system (14) is asymptotically stable via the output feedback PD gains*

(16) $$\begin{array}{c} \begin{array}{cc} \; & K_{p}=X{\tilde{P}}^{-1}C^{T}{\left(\;CC^{T}\right)}^{-1},\\ \; & K_{d}=Y{\tilde{P}}^{-1}C^{T}{\left(\;CC^{T}\right)}^{-1}, \end{array} \end{array}$$

*and the guaranteed cost value can be calculated as*

(17) $$\begin{array}{c} J^{*}=\lambda_{\max}\left({\tilde{P}}^{-1}\right){\left(∥\phi ∥\right)}^{2}. \end{array}$$

**Proof.**  Let us consider the Lyapunov function

(18) $$V\left(\vartheta \left(t\right)\right)=\vartheta^{T}\left(t\right)P\vartheta \left(t\right).$$

□

Its fractional derivative along with the system trajectory (9) using Lemma 3 is

(19) $$\begin{array}{c} \begin{array}{cc} \; & {}^{C}D_{}^{q}V\left(\vartheta \left(t\right)\right)+\vartheta^{T}\left(t\right)Q_{1}\vartheta \left(t\right)+\\ & {\left(K_{p}C\vartheta \left(t-\delta \right)+K_{d}C{\;}^{C}D_{}^{q}\vartheta \left(t-\delta \right)\right)}^{T}Q_{2}\left(K_{p}C\vartheta \left(t-\delta \right)+K_{d}C{\;}^{C}D_{}^{q}\vartheta \left(t-\delta \right)\right)\\ \; & \leq {\left(\vartheta^{T}\left(t\right)P\right)}^{C}D_{}^{q}\vartheta \left(t\right)\;+{{(}^{C}D_{}^{q}\vartheta \left(t\right))}^{T}P\vartheta \left(t\right)+\vartheta^{T}\left(t\right)Q_{1}\vartheta \left(t\right)\\ \; & \;\;\;+(K_{p}C\vartheta \left(t-\delta \right)+K_{d}C{\;}^{C}D_{}^{q}\vartheta \left(t-\delta \right){)}^{T}Q_{2}\left(K_{p}C\vartheta \left(t-\delta \right)+K_{d}C{\;}^{C}D_{}^{q}\vartheta \left(t-\delta \right)\right)\\ \; & \leq \vartheta^{T}\left(t\right)\;\left(PA+A^{T}P\right)\;\vartheta \left(t\right)+\vartheta^{T}\left(t\right)\;\left(P\Delta A+\Delta A^{T}P\right)\;\vartheta \left(t\right)\\ \; & \;\;\;+\vartheta^{T}\left(t\right)\;\left(PA_{d}+P\Delta A_{d}+PBK_{p}C+P\Delta BK_{p}C\right)\vartheta \left(t-\delta \right)\\ \; & \;\;\;+\vartheta^{T}\left(t-\delta \right)\;\left({A_{d}}^{T}P+C^{T}{K_{p}}^{T}B^{T}P+C^{T}{K_{p}}^{T}\Delta B^{T}P+\Delta {A_{d}}^{T}P\right)\vartheta \left(t\right)\\ \; & \;\;\;+{\;}^{C}D_{}^{q}\vartheta^{T}\left(t-\delta \right)\;\left({A_{0}}^{T}P+C^{T}{K_{d}}^{T}B^{T}P+\Delta {A_{0}}^{T}P+C^{T}{K_{d}}^{T}\Delta B^{T}P\right)\vartheta \left(t\right)\\ \; & \;\;\;+\vartheta^{T}\left(t\right)\;\left(PA_{0}+P\Delta A_{0}+PBK_{d}C+P\Delta BK_{d}C\right){\;}^{C}D_{}^{q}\vartheta \left(t-\delta \right)\\ \; & \;\;\;+\vartheta^{T}\left(t\right)Q_{1}\vartheta \left(t\right)+\vartheta^{T}\left(t-\delta \right){\left(K_{p}C\right)}^{T}Q_{2}\left(K_{d}C\right){\;}^{C}D_{}^{q}\vartheta \left(t-\delta \right)\\ \; & \;\;\;+\vartheta^{T}\left(t-\delta \right){\left(K_{p}C\right)}^{T}Q_{2}\left(K_{p}C\right)\;\vartheta \left(t-\delta \right){+}^{C}D_{}^{q}\vartheta^{T}\left(t-\delta \right)C^{T}{K_{d}}^{T}Q_{2}K_{d}C{\;}^{C}D_{}^{q}\vartheta \left(t-\delta \right)\\ \; & \;\;\;+{\;}^{C}D^{q}\vartheta^{T}\left(t-\delta \right)C^{T}{K_{d}}^{T}Q_{2}K_{p}C\vartheta \left(t-\delta \right). \end{array} \end{array}$$

Based on Lemma 5, we can get

(20) $$\begin{array}{c} \begin{array}{cc} \; & \vartheta^{T}\left(t-\delta \right)\;{\left(K_{p}C\right)}^{T}Q_{2}\left(K_{d}C\right)\;{}^{C}D_{}^{q}\vartheta \left(t-\delta \right)+{}^{C}D_{}^{q}\vartheta^{T}\left(t-\delta \right)\;C^{T}{K_{d}}^{T}Q_{2}K_{p}C\;\vartheta \left(t-\delta \right)\\ \; & \leq \vartheta^{T}\left(t-\delta \right)\;C^{T}{K_{P}}^{T}\Sigma K_{p}C\;\vartheta \left(t-\delta \right)+{}^{C}D_{}^{q}\vartheta^{T}\left(t-\delta \right)\;C^{T}{K_{d}}^{T}{Q_{2}}^{T}\Sigma^{-1}Q_{2}K_{d}C\;{}^{C}D_{}^{q}\vartheta \left(t-\delta \right). \end{array} \end{array}$$

Considering (18) and using Lemma 4, whenever ϑ(t) satisfies

(21) $$\begin{array}{c} \sigma V(t,\vartheta (t))>V(t+\varrho ,\vartheta (t+\varrho )),\;\;-\delta \leq \varrho \leq 0, \end{array}$$

we can describe for some σ>1

(22) $$\begin{array}{c} -\vartheta^{T}\left(t-\delta \right)P\vartheta \left(t-\delta \right)+\sigma \vartheta^{T}\left(t\right)P\vartheta \left(t\right)\geq 0. \end{array}$$

From (14), we have the following null equation

$$\begin{array}{cc} \; & 2\left({}^{C}D_{}^{q}\vartheta^{T}\left(t\right)\;-{}^{C}D_{}^{q}\vartheta^{T}\left(t-\delta \right)\right)P\left({-}^{C}D_{}^{q}\vartheta \left(t\right)+\left(A+\Delta A\right)\vartheta \left(t\right)+(A_{d}+\Delta A_{d}\right.\\ \; & \;\left.+BK_{p}C+\Delta BK_{p}C)\vartheta \left(t-\delta \right)+{\left(A_{0}+\Delta A_{0}+BK_{d}C+\Delta BK_{d}C\right)}^{C}D_{}^{q}\vartheta \left(t-\delta \right)\right)=0. \end{array}$$

Combining (19) with (20) and (22) as well as using the above expression yields

(23) $$\begin{array}{c} \begin{array}{cc} \; & {}^{C}D_{}^{q}V\left(\vartheta \left(t\right)\right)+\vartheta^{T}\left(t\right)Q_{1}\vartheta \left(t\right)+{\left(K_{p}C\vartheta \left(t-\delta \right)+K_{d}C{\;}^{C}D_{}^{q}\vartheta \left(t-\delta \right)\right)}^{T}Q_{2}(K_{p}C\vartheta \left(t-\delta \right)\\ \; & \;\;+K_{d}C{\;}^{C}D_{}^{q}\vartheta \left(t-\delta \right))\\ \; & \leq \vartheta^{T}\left(t\right)\;\left(PA+A^{T}P+Q_{1}+\sigma P\right)\;\vartheta \left(t\right)+\vartheta^{T}\left(t\right)\;\left(P\Delta A+\Delta A^{T}P\right)\;\vartheta \left(t\right)\\ \; & \;\;+\vartheta^{T}\left(t\right)\;\left(PA_{d}+P\Delta A_{d}+PBK_{p}C+P\Delta BK_{p}C\right)\vartheta \left(t-\delta \right)\\ \; & \;\;+\vartheta^{T}\left(t-\delta \right)\;\left({A_{d}}^{T}P+\Delta {A_{d}}^{T}P+C^{T}{K_{p}}^{T}B^{T}P+C^{T}{K_{p}}^{T}\Delta B^{T}P\right)\vartheta \left(t\right)\\ \; & \;\;+2\vartheta^{T}\left(t\right)\;\left(PA_{0}+P\Delta A_{0}+PBK_{d}C+P\Delta BK_{d}C\right){\;}^{C}D_{}^{q}\vartheta \left(t-\delta \right)\\ \; & \;\;+\vartheta^{T}\left(t-\delta \right)\;\left(C^{T}{K_{p}}^{T}Q_{2}K_{p}C+C^{T}{K_{p}}^{T}\Sigma K_{p}C-P\right)\vartheta \left(t-\delta \right)\\ \; & \;\;+{\;}^{C}D_{}^{q}\vartheta^{T}\left(t-\delta \right)\;\left(C^{T}{K_{d}}^{T}Q_{2}K_{d}C+C^{T}{K_{d}}^{T}{Q_{2}}^{T}\Sigma^{-1}Q_{2}K_{d}C\right){\;}^{C}D_{}^{q}\vartheta \left(t-\delta \right)\\ \; & \;\;-2\;{}^{C}D_{}^{q}\vartheta^{T}\left(t\right)P{}^{C}D_{}^{q}\vartheta \left(t\right)+2\;{}^{C}D_{}^{q}\vartheta^{T}\left(t\right)\left(PA+P\Delta A\right)\vartheta \left(t\right)\\ \; & \;\;-2\;{}^{C}D_{}^{q}\vartheta^{T}\left(t-\delta \right)\left(PA_{d}+P\Delta A_{d}+PBK_{p}C+P\Delta BK_{p}C\right)\vartheta \left(t-\delta \right)\\ \; & \;\;-2\;{}^{C}D_{}^{q}\vartheta^{T}\left(t-\delta \right)\left(PA_{0}+P\Delta A_{0}+PBK_{d}C+P\Delta BK_{d}C\right)\;{}^{C}D_{}^{q}\vartheta \left(t-\delta \right)\\ \; & \;\;+2\;{}^{C}D_{}^{q}\vartheta^{T}\left(t\right)\left(PA_{d}+P\Delta A_{d}+PBK_{p}C+P\Delta BK_{p}C\right)\vartheta \left(t-\delta \right)\\ \; & \;\;+2\;{}^{C}D_{}^{q}\vartheta^{T}\left(t\right)\left(PA_{0}+P\Delta A_{0}+PBK_{d}C+P\Delta BK_{d}C\right){\;}^{C}D_{}^{q}\vartheta \left(t-\delta \right)\\ \; & \;\;+2\;{}^{C}D_{}^{q}\vartheta^{T}\left(t-\delta \right)P\;{}^{C}D_{}^{q}\vartheta \left(t\right)-2\;{}^{C}D_{}^{q}\vartheta^{T}\left(t-\delta \right)\left(PA+P\Delta A\right)\vartheta \left(t\right)\\ \; & \leq \eta^{T}\left(t\right)\;\psi \eta \;\left(t\right). \end{array} \end{array}$$

Here,

$$\begin{array}{c} \psi =\left(\begin{array}{cccc} \psi_{11} & \psi_{12} & \psi_{13} & \psi_{14}\\ {}* & \psi_{22} & \psi_{23} & \psi_{24}\\ {}* & * & \psi_{33} & \psi_{34}\\ {}* & * & * & \psi_{44} \end{array}\right)<0, \end{array}$$

where

$$\begin{array}{c} \begin{array}{cc} \; & \psi_{11}=PA+A^{T}P+Q_{1}+\sigma P+P\Delta A+\Delta A^{T}P,\\ \; & \psi_{12}=PA_{d}+P\Delta A_{d}+PBK_{p}C+P\Delta BK_{p}C,\\ \; & \psi_{13}=PA_{0}+P\Delta A_{0}+PBK_{d}C+P\Delta BK_{d}C-A^{T}P-\Delta A^{T}P,\\ \; & \psi_{14}=A^{T}P+\Delta A^{T}P,\\ \; & \psi_{22}={\left(K_{p}C\right)}^{T}Q_{2}\left(K_{p}C\right)\;+{\left(K_{p}C\right)}^{T}\Sigma \left(K_{p}C\right)\;-P,\\ \; & \psi_{23}=-{A_{d}}^{T}P-\Delta {A_{d}}^{T}P-C^{T}{K_{p}}^{T}B^{T}P-C^{T}{K_{p}}^{T}\Delta B^{T}P,\\ \; & \psi_{24}={A_{d}}^{T}P+\Delta {A_{d}}^{T}P+C^{T}{K_{p}}^{T}B^{T}P+C^{T}{K_{p}}^{T}\Delta B^{T}P,\\ \; & \psi_{33}=-2\left(PA_{0}+P\Delta A_{0}+PBK_{d}C+P\Delta BK_{d}C\right)+\\ & {\left(K_{d}C\right)}^{T}Q_{2}\left(K_{d}C\right)+{\left(K_{d}C\right)}^{T}{Q_{2}}^{T}\Sigma^{-1}Q_{2}\left(K_{d}C\right),\\ \; & \psi_{34}={A_{0}}^{T}P+\Delta {A_{0}}^{T}P+C^{T}{K_{d}}^{T}B^{T}P+C^{T}{K_{d}}^{T}\Delta B^{T}P+P,\\ \; & \psi_{44}=-2P, \end{array} \end{array}$$

and $\eta^{T}\left(t\right)=\left[\begin{array}{cccc} \vartheta^{T}\left(t\right), & \vartheta^{T}\left(t-\delta \right), & {}^{C}D_{}^{q}\vartheta^{T}\left(t-\delta \right), & {}^{C}D_{}^{q}\vartheta^{T}\left(t\right) \end{array}\right]$.

The above inequality, i.e., ψ≤0, can be decomposed as

(24) $$\begin{array}{c} \begin{array}{cc} \; & \underset{\Omega }{\underbrace{\left(\begin{array}{cccc} \Omega_{11} & \Omega_{12} & \Omega_{13} & A^{{}^{T}}P\\ {}* & \Omega_{22} & \Omega_{23} & \Omega_{24}\\ {}* & * & \Omega_{33} & \Omega_{34}\\ {}* & * & * & -2P \end{array}\right)}}+\left(\begin{array}{c} PH\\ 0\\ -PH\\ PH \end{array}\right)M\left(t\right)\left(\begin{array}{cccc} E_{0} & E_{1}+E_{3}K_{p}C & E_{2}+E_{3}K_{d}C & 0 \end{array}\right)\\ \; & \;+\left(\begin{array}{c} {E_{0}}^{T}\\ {E_{1}}^{T}+C^{T}{K_{p}}^{T}{E_{3}}^{T}\\ {E_{2}}^{T}+C^{T}{K_{d}}^{T}{E_{3}}^{T}\\ 0 \end{array}\right)M^{T}\left(t\right)\left(\begin{array}{cccc} H^{T}P & 0 & -H^{T}P & H^{T}P \end{array}\right)<0 \end{array} \end{array}$$

where

$$\begin{array}{c} \begin{array}{cc} \; & \Omega_{11}=PA+A^{T}P+\sigma P+Q_{1},\\ \; & \Omega_{12}=PA_{d}+PBK_{p}C\;\;,\\ \; & \Omega_{13}=PA_{0}+PBK_{d}C-A^{T}P,\\ \; & \Omega_{22}={\left(K_{p}C\right)}^{T}Q_{2}\left(K_{p}C\right)+{\left(K_{p}C\right)}^{T}\Sigma \left(K_{p}C\right)-P,\\ \; & \Omega_{23}=-{A_{d}}^{T}P-C^{T}{K_{p}}^{T}B^{T}P,\\ \; & \Omega_{24}={A_{d}}^{T}P+C^{T}{K_{p}}^{T}B^{T}P,\\ \; & \Omega_{33}={\left(K_{d}C\right)}^{T}Q_{2}\left(K_{d}C\right)+{\left(K_{d}C\right)}^{T}{Q_{2}}^{T}\Sigma^{-1}Q_{2}\left(K_{d}C\right)-2\left(PA_{0}+PBK_{d}C\right),\\ \; & \Omega_{34}={A_{0}}^{T}P+C^{T}{K_{d}}^{T}B^{T}P+P. \end{array} \end{array}$$

Note that following Lemma 2, inequality (24) is further equivalent to

(25) $$\begin{array}{c} \begin{array}{cc} \; & \Omega +\lambda \left(\begin{array}{c} PH\\ 0\\ -PH\\ PH \end{array}\right)\left(\begin{array}{cccc} H^{T}P & 0 & -H^{T}P & H^{T}P \end{array}\right)\\ \; & \;+\lambda^{-1}\left(\begin{array}{c} {E_{0}}^{T}\\ {E_{1}}^{T}+C^{T}{K_{p}}^{T}{E_{3}}^{T}\\ {E_{2}}^{T}+C^{T}{K_{d}}^{T}{E_{3}}^{T}\\ 0 \end{array}\right)\left(\begin{array}{cccc} E_{0} & E_{1}+E_{3}K_{p}C & E_{2}+E_{3}K_{d}C & 0 \end{array}\right)<0. \end{array} \end{array}$$

Using Lemma 1 results in

(26) $$\begin{array}{c} \left(\begin{array}{ccccccccccc} \phi_{11} & \phi_{12} & \phi_{13} & A^{T}P & {E_{0}}^{T} & \lambda PH & Q_{1} & 0 & 0 & 0 & 0\\ {}* & -P & \phi_{23} & \phi_{24} & \phi_{25} & 0 & 0 & C^{T}{K_{p}}^{T} & C^{T}{K_{p}}^{T} & 0 & 0\\ {}* & * & \phi_{33} & \phi_{34} & \phi_{35} & -\lambda PH & 0 & 0 & 0 & C^{T}{K_{d}}^{T} & C^{T}{K_{d}}^{T}{Q_{2}}^{T}\\ {}* & * & * & -2P & 0 & \lambda PH & 0 & 0 & 0 & 0 & 0\\ {}* & * & * & * & -\lambda I & 0 & 0 & 0 & 0 & 0 & 0\\ {}* & * & * & * & * & -\lambda I & 0 & 0 & 0 & 0 & 0\\ {}* & * & * & * & * & * & -Q_{1} & 0 & 0 & 0 & 0\\ {}* & * & * & * & * & * & * & -{Q_{2}}^{-1} & 0 & 0 & 0\\ {}* & * & * & * & * & * & * & * & -\Sigma^{-1} & 0 & 0\\ {}* & * & * & * & * & * & * & * & * & -{Q_{2}}^{-1} & 0\\ {}* & * & * & * & * & * & * & * & * & * & -\Sigma \end{array}\right), \end{array}$$

where

$$\begin{array}{c} \begin{array}{cc} \; & \phi_{11}=PA+P+A^{T}P,\;\;\phi_{12}=PA_{d}+PBK_{p}C,\;\;\phi_{13}=PA_{0}+PBK_{d}C-A^{T}P,\\ \; & \phi_{23}=-{A_{d}}^{T}P-C^{T}{K_{p}}^{T}B^{T}P,\;\;\phi_{24}={A_{d}}^{T}P+C^{T}{K_{p}}^{T}B^{T}P,\;\;\phi_{25}={E_{1}}^{T}+C^{T}{K_{p}}^{T}{E_{3}}^{T},\\ \; & \phi_{33}=-2\left(PA_{0}+PBK_{d}C\right),\;\;\phi_{34}={A_{0}}^{T}P+C^{T}{K_{d}}^{T}B^{T}P+P,\;\;\phi_{35}={E_{2}}^{T}+C^{T}{K_{d}}^{T}{E_{3}}^{T}. \end{array} \end{array}$$

Pre- and postmultiplying the matrix ϕ given in (26) by $diag\left\{P^{-1},P^{-1},P^{-1},P^{-1},I,I,I,I,I,I,I\right\}$ and considering $P^{-1}=\tilde{P}$, σ>1, $X=K_{p}C\tilde{P}$, and $Y=K_{d}C\tilde{P}$, it gives

(27) $$\begin{array}{c} \Theta =\left(\begin{array}{ccccccccccc} \Theta_{11} & \Theta_{12} & \Theta_{13} & \tilde{P}A^{T} & \tilde{P}{E_{0}}^{T} & \lambda H & \tilde{P}Q_{1} & 0 & 0 & 0 & 0\\ {}* & -\tilde{P} & \Theta_{23} & \Theta_{24} & \Theta_{25} & 0 & 0 & X^{T} & X^{T} & 0 & 0\\ {}* & * & \Theta_{33} & \Theta_{34} & \Theta_{35} & -\lambda H & 0 & 0 & 0 & Y^{T} & Y^{T}{Q_{2}}^{T}\\ {}* & * & * & -2\tilde{P} & 0 & \lambda H & 0 & 0 & 0 & 0 & 0\\ {}* & * & * & * & -\lambda I & 0 & 0 & 0 & 0 & 0 & 0\\ {}* & * & * & * & * & -\lambda I & 0 & 0 & 0 & 0 & 0\\ {}* & * & * & * & * & * & -Q_{1} & 0 & 0 & 0 & 0\\ {}* & * & * & * & * & * & * & -Q_{2}^{-1} & 0 & 0 & 0\\ {}* & * & * & * & * & * & * & * & -\Sigma^{-1} & 0 & 0\\ {}* & * & * & * & * & * & * & * & * & -Q_{2}^{-1} & 0\\ {}* & * & * & * & * & * & * & * & * & * & -\Sigma \end{array}\right)<0, \end{array}$$

where

$$\begin{array}{c} \begin{array}{cc} \; & \Theta_{11}=A\tilde{P}+\tilde{P}A^{T}+\tilde{P},\;\;\Theta_{12}=A_{d}\tilde{P}+BX,\;\;\Theta_{13}=A_{0}\tilde{P}+BY-\tilde{P}A^{T},\\ \; & \Theta_{23}=-\tilde{P}{A_{d}}^{T}-X^{T}B^{T},\;\;\Theta_{24}=\tilde{P}{A_{d}}^{T}+X^{T}B^{T},\;\;\Theta_{25}=\tilde{P}{E_{1}}^{T}+X^{T}{E_{3}}^{T},\\ \; & \Theta_{33}=-2\left(A_{0}\tilde{P}+BY\right),\;\;\Theta_{34}=\tilde{P}+\tilde{P}{A_{0}}^{T}+Y^{T}B^{T},\;\;\Theta_{35}=\tilde{P}{E_{2}}^{T}+Y^{T}{E_{3}}^{T}. \end{array} \end{array}$$

Applying Lemma 4, the overall system in (14) using the PD controller in (12) is asymptotically stable. Moreover, we have

(28) $$\begin{array}{c} \begin{array}{cc} {}^{C}D_{}^{q}\left(\vartheta^{T}\left(t\right)P\vartheta \left(t\right)\right) & \leq -\vartheta^{T}\left(t\right)\;Q_{1}\;\vartheta \left(t\right)-u^{T}\left(t\right)Q_{2}u\;\left(t\right)\\ & \leq 0. \end{array} \end{array}$$

Taking the integral of order *q* on both sides of (28) gives

(29) J(u)≤V(0,ϑ(0))−V(h,ϑ(h)).

Since V(h,ϑ(h))≥0, it yields

(30) $$\begin{array}{cc} J(u) & \leq V(0,\vartheta (0))-V(h,\vartheta (h))\\ & \leq V(0,\vartheta (0))\\ & \leq \lambda_{\max}\left({\tilde{P}}^{-1}\right){\left(∥\phi ∥\right)}^{2}\\ & =J^{*}, \end{array}$$

which ends the proof.

**Remark** **1.** *There is no restriction to apply the main results for the case of large-scale matrices, especially the practical application. For more information regarding the computational complexity of differential equations with FO, please see [63].*

## 5. Application

In this section, we adopt as a case study a two-stage chemical reactor in order to discuss how the proposed methodology can be related to a specific application. The FO description of the reactor system [64] is given by

(31) $$\begin{array}{cc} \; & \nu_{1}{}^{C}D_{t}^{q}c_{1}=f_{1}c_{1f}\left(t\right)+rc_{2}\left(t-\delta \right)+f_{d}c_{d}\left(t\right)-\left(f_{1}+r+f_{d}\right)c_{1}\left(t\right)-\nu_{1}\left(\kappa_{1}+\Delta \kappa_{1}\left(t\right)\right)c_{1}\left(t\right),\\ \; & \nu_{2}{}^{C}D_{t}^{q}c_{2}=\left(f_{d}+f_{1}-f_{p1}+r\right)c_{1}\left(t\right)+f_{2}c_{2f}\left(t\right)-\left(f_{p2}+r\right)c_{2}\left(t\right)-\nu_{2}\left(\kappa_{2}+\kappa_{2}\left(t\right)\right)c_{2}\left(t\right), \end{array}$$

where $f_{1}$ and $f_{2}$ represent the feed rates, $c_{1f}$ and $c_{2f}$ denote the reactor’s feed composition, and $f_{d}$ represents the disturbance to an extra feed stream with a composition $c_{d}$. Furthermore, the recycle flow rate is denoted by *r*, the reactors volumes are represented by $\nu_{1}$ and $\nu_{2}$, and $\Delta \kappa_{1}$ and $\Delta \kappa_{2}$ stand for the system uncertainties, which are time-varying. In the real world, the parameters are unknown, but we can assume the upper bound on their values. Defining the reactor residence times $\theta_{1}$ and $\theta_{2}$ as

$$\theta_{1}=\frac{\nu_{1}}{f_{1}+r+f_{d}}\;,\;\theta_{2}=\frac{\nu_{2}}{f_{p2}+r},$$

the state-space representation of Equation (31) can be written by

(32) $${}^{C}D_{t}^{q}\vartheta \left(t\right)=\left(A_{d}+\Delta A_{d}\right)\vartheta \left(t-\delta \right)+\left(A+\Delta A\right)\vartheta \left(t\right)+\left(B+\Delta B\right)u\left(t\right),$$

with

(33) $$\begin{array}{cc} \; & A=\left[\begin{array}{cc} -(\frac{1}{\theta_{1}}+\kappa_{1}) & 0\\ \frac{f_{p2}-f_{2}+r}{\nu_{2}} & -(\frac{1}{\theta_{2}}+\kappa_{2}) \end{array}\right],\;A{}_{d}\left(t\right)=\left[\begin{array}{cc} 0 & \frac{r}{f_{1}}\\ 0 & 0 \end{array}\right],\;B=\left(\begin{array}{cc} \frac{f_{1}}{\nu_{1}} & 0\\ 0 & \frac{f_{2}}{\nu_{2}} \end{array}\right), \end{array}$$

which is a special case of (9) with $A_{0}=\Delta A_{0}=0$.

The next theorem provides a criterion to stabilize the system (32).

**Theorem** **2.** *For given symmetric positive-definite matrices $Q_{i}\;\left(i=1,2\right)$ in (11), if there exist a symmetric and positive-definite matrix $\tilde{P}$, matrices X and Y with compatible dimension, a non-negative scalar λ, and a diagonal positive matrix Σ satisfying*

(34) $$\begin{array}{c} \left(\begin{array}{ccccccccccc} \Theta_{11} & \Theta_{12} & BY-\tilde{P}A^{T} & \tilde{P}A^{T} & \tilde{P}{E_{0}}^{T} & \lambda H & \tilde{P}Q_{1} & 0 & 0 & 0 & 0\\ {}* & -\tilde{P} & \Theta_{23} & \Theta_{24} & \Theta_{25} & 0 & 0 & X^{T} & X^{T} & 0 & 0\\ {}* & * & -2BY & \tilde{P}+Y^{T}B^{T} & Y^{T}{E_{3}}^{T} & -\lambda H & 0 & 0 & 0 & Y^{T} & Y^{T}{Q_{2}}^{T}\\ {}* & * & * & -2\tilde{P} & 0 & \lambda H & 0 & 0 & 0 & 0 & 0\\ {}* & * & * & * & -\lambda I & 0 & 0 & 0 & 0 & 0 & 0\\ {}* & * & * & * & * & -\lambda I & 0 & 0 & 0 & 0 & 0\\ {}* & * & * & * & * & * & -Q_{1} & 0 & 0 & 0 & 0\\ {}* & * & * & * & * & * & * & -Q_{2}^{-1} & 0 & 0 & 0\\ {}* & * & * & * & * & * & * & * & -\Sigma^{-1} & 0 & 0\\ {}* & * & * & * & * & * & * & * & * & -Q_{2}^{-1} & 0\\ {}* & * & * & * & * & * & * & * & * & * & -\Sigma \end{array}\right)<0 \end{array}$$

*where $\Theta_{11}=A\tilde{P}+\tilde{P}A^{T}+\tilde{P}$, $\Theta_{12}=A_{d}\tilde{P}+BX$, $\Theta_{23}=-\tilde{P}{A_{d}}^{T}-X^{T}B^{T},$ $\Theta_{24}=\tilde{P}{A_{d}}^{T}+X^{T}B^{T},$ and $\Theta_{25}=\tilde{P}{E_{1}}^{T}+X^{T}{E_{3}}^{T}$, then the overall system is asymptotically stable with the controller matrices*

(35) $$\begin{array}{c} \begin{array}{cc} \; & K_{p}=XP^{-1}C^{T}{\left(\;CC^{T}\right)}^{-1},\\ \; & K_{d}=YP^{-1}C^{T}{\left(\;CC^{T}\right)}^{-1}, \end{array} \end{array}$$

and $J^{*}=\lambda_{\max}\left({\tilde{P}}^{-1}\right){\left(∥\phi ∥\right)}^{2}.$

**Proof.**  It suffices to perform steps similar to the ones provided in the Proof of Theorem 1, considering $A_{0}=\Delta A_{0}=0$. Therefore, the proof is completed. □

## 6. Simulation Results

Here, we verified the capability of the output feedback PD controller, where the modified Adams–Bashforth–Moulton algorithm [65] was employed to solve the FO differential equations in MATLAB software.

**Example** **1.** *Consider an FO system (14) with parameters*

$$\begin{array}{cc} \; & A=\left(\begin{array}{cc} -0.5 & 0.8\\ -0.6 & -0.9 \end{array}\right),\;A_{d}=\left(\begin{array}{cc} 0.1 & 0\\ -0.5 & 0.3 \end{array}\right),\;A_{0}=\left(\begin{array}{cc} -0.2 & 0.5\\ 0 & 0.3 \end{array}\right),\;B=\left(\begin{array}{cc} -0.8 & -0.2\\ 0.1 & 0.5 \end{array}\right),\\ & \;H={\left(\begin{array}{cc} -0.1 & -0.1 \end{array}\right)}^{T},\;C=\left(\begin{array}{cc} 1 & 0 \end{array}\right),\;E_{3}=\left(\begin{array}{cc} 0.3 & 0.1 \end{array}\right),\;E_{2}=\left(\begin{array}{cc} -0.3 & 0.2 \end{array}\right),\;E_{1}=\left(\begin{array}{cc} 0.5 & 0.2 \end{array}\right),\\ & E_{0}=\left(\begin{array}{cc} 0.5 & 0.1 \end{array}\right). \end{array}$$

*By choosing $Q_{1}=I_{2\times 2}$, $Q_{2}=2$, and δ=0.2, we have*

$$\begin{array}{cc} \; & \tilde{P}=\left(\begin{array}{cc} 0.5025 & 0.0006\\ 0.0006 & 0.4024 \end{array}\right),\;\;X=\left(\begin{array}{cc} -0.0257 & -0.0130\\ 0.01437 & -0.0665 \end{array}\right),\\ \; & Y=\left(\begin{array}{cc} -0.1788 & 0.3106\\ 0.0813 & 0.1239 \end{array}\right),\;\lambda =4.4465. \end{array}$$

**Figure 1:**
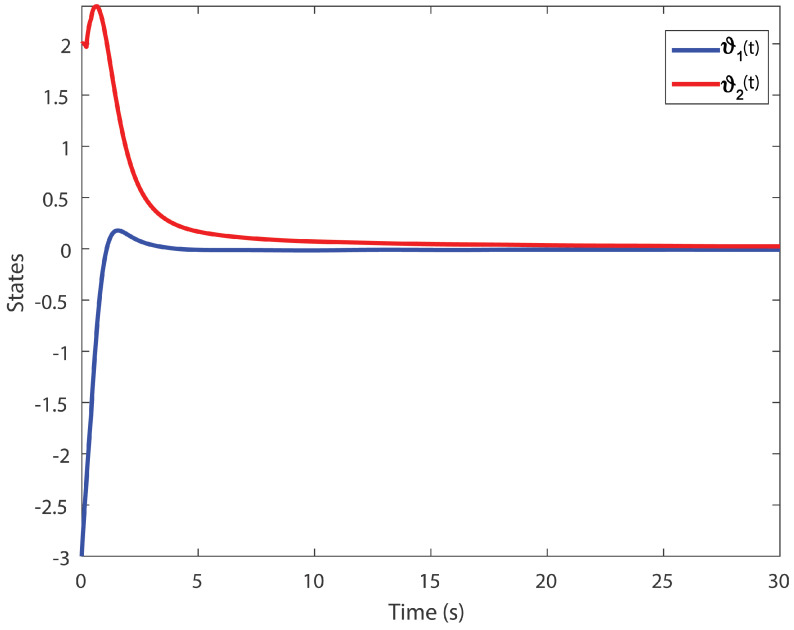
Time evolution for Example 1 with q=0.9.

**Figure 2:**
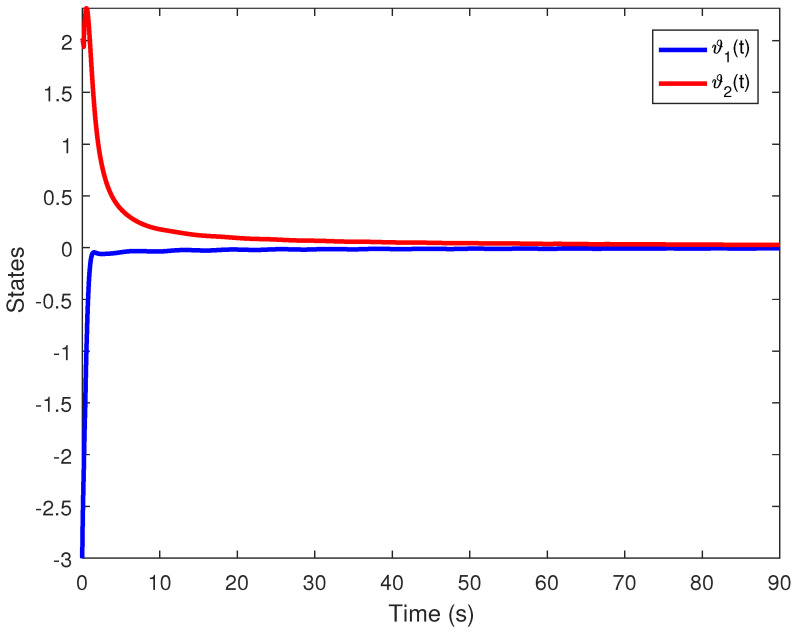
Time evolution for Example 1 with q=0.8.

**Figure 3:**
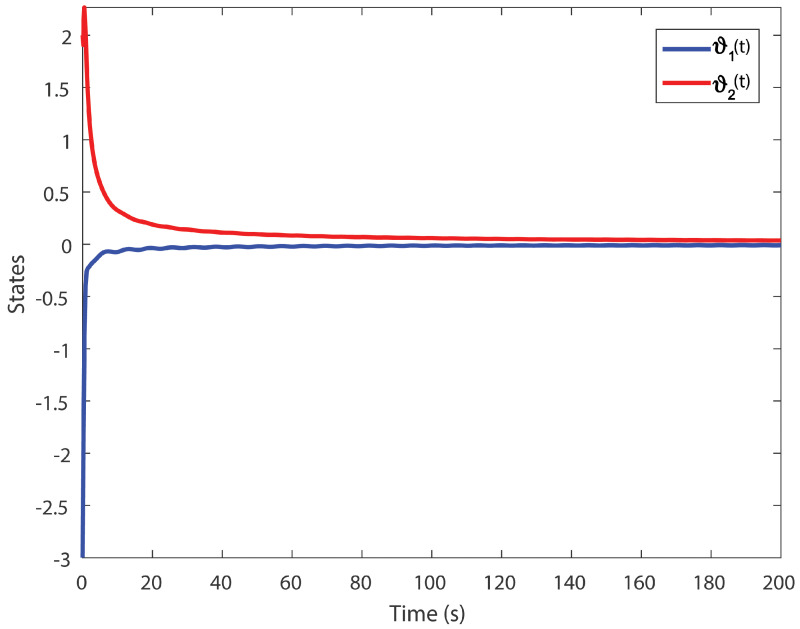
Time evolution for Example 1 with q=0.7.

**Figure 4:**
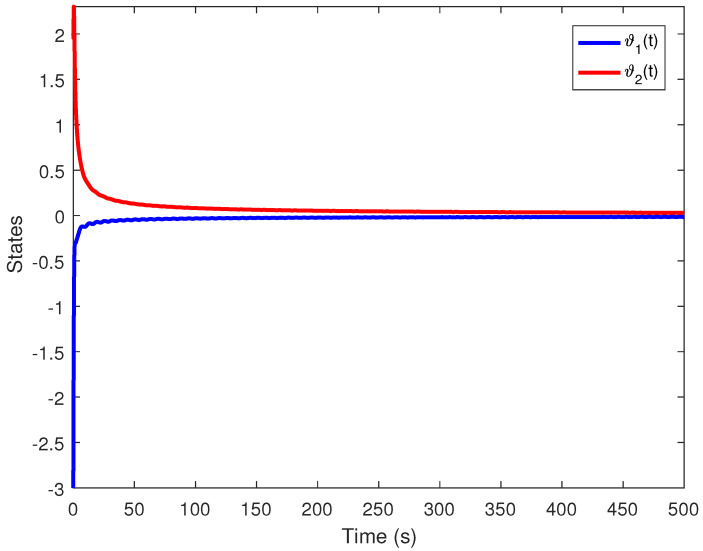
Time evolution for Example 1 with q=0.6.

We obtain the PD controller gains as $K_{P}={\left(0.0511\;\;\;0.2862\right)}^{T}$ and $K_{d}={\left(-0.3569\;\;\;0.1614\right)}^{T}$. The minimum upper bound on (11) is $J^{*}=32.3050$. Figure 1, Figure 2, Figure 3 and Figure 4 illustrate the time evolution of the overall system for distinct fractional order values, i.e., q=0.9,0.8,0.7,0.6. According to the results, we can infer that the system’s behavior is satisfactory. Moreover, we conclude that decreasing the value of *q* results in a larger settling time.

**Example** **2.** *Considering the two-stage chemical reactor model (31) with $f_{1}=0.4,f_{2}=0.5,\nu_{1}=\nu_{2}=1,\kappa_{1}=\kappa_{2}=1,f_{p1}=f_{p2}=0.5,r=0.25,f_{d}=0.1,\Delta_{1}=0.4,\Delta_{2}=0.5,\theta_{1}=0.75$, and $\theta_{2}=0.5$ [64], we get*

$$\begin{array}{c} A=\left(\begin{array}{cc} -1.75 & 0\\ 0.25 & -1.75 \end{array}\right),\;A_{d}=\left(\begin{array}{cc} 0 & 0.25\\ 0 & 0 \end{array}\right),\;B=\left(\begin{array}{cc} 0.4 & 0\\ 0 & 0.5 \end{array}\right), \end{array}$$

$$\begin{array}{cc} \; & C=\left(\begin{array}{cc} 1 & 0 \end{array}\right),\;H=\left(\begin{array}{c} 1\\ 1 \end{array}\right),\;E_{0}=\left(\begin{array}{cc} -0.6 & -0.6 \end{array}\right),\;E_{1}=\left(\begin{array}{cc} -0.5 & 0.2 \end{array}\right),\;E_{3}=\left(\begin{array}{cc} -0.3 & 0.1 \end{array}\right). \end{array}$$

*By choosing $Q_{1}=I_{2\times 2},\;Q_{2}=1,$ and δ=1, and considering Theorem 2, we obtain*

$$\begin{array}{cc} \; & \tilde{P}=\left(\begin{array}{cc} 0.4580 & -0.0107\\ -0.0107 & 0.4603 \end{array}\right),\;X=\left(\begin{array}{cc} 0.0769 & -0.1274\\ 0.0140 & -0.0147 \end{array}\right),\\ \; & Y=\left(\begin{array}{cc} 0.1134 & 0.0280\\ 0.0047 & 0.1258 \end{array}\right),\;\lambda =0.3562. \end{array}$$

The gains of the PD control law are $K_{P}={\left(\begin{array}{cc} 0.1614, & 0.0298 \end{array}\right)}^{T}$ and $K_{d}={\left(\begin{array}{cc} 0.2492, & 0.0167 \end{array}\right)}^{T}$ and the minimum upper bound on (11) is $J^{*}=7.2474$.

**Figure 5:**
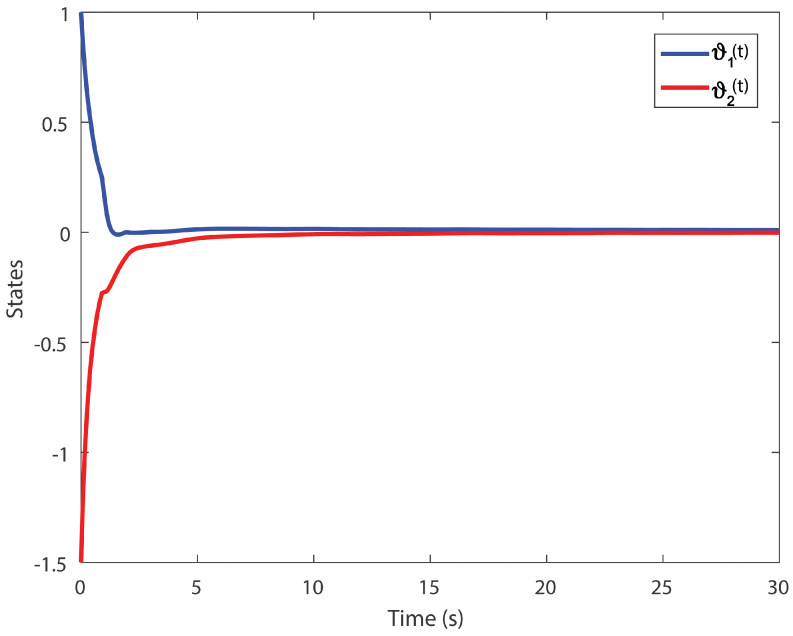
Time evolution of two-stage chemical reactor system with q=0.9.

**Figure 6:**
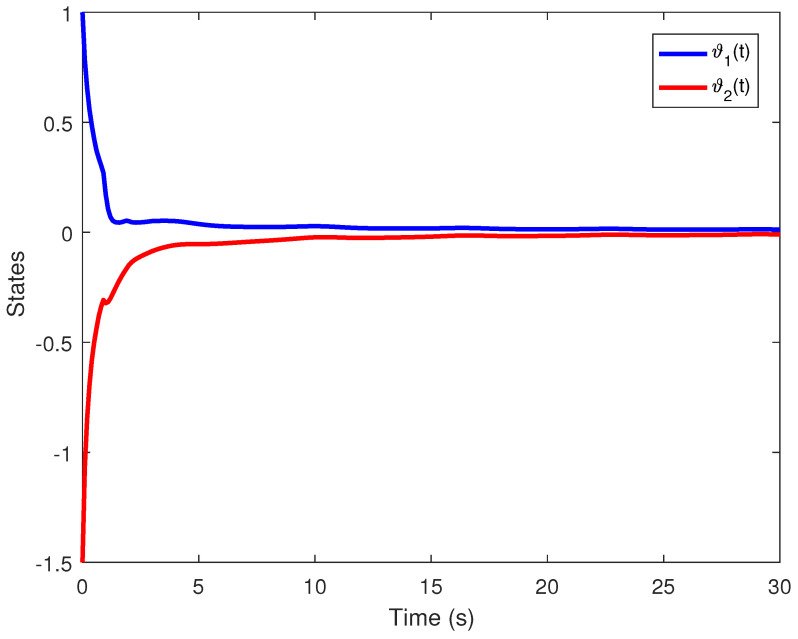
Time evolution for Example 2 with q=0.8.

**Figure 7:**
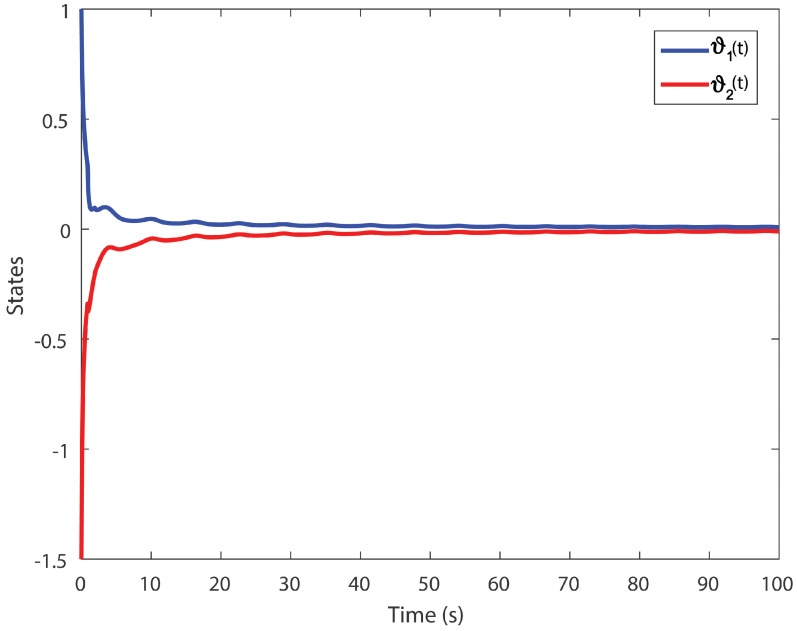
Time evolution of two-stage chemical reactor system with q=0.7.

**Figure 8:**
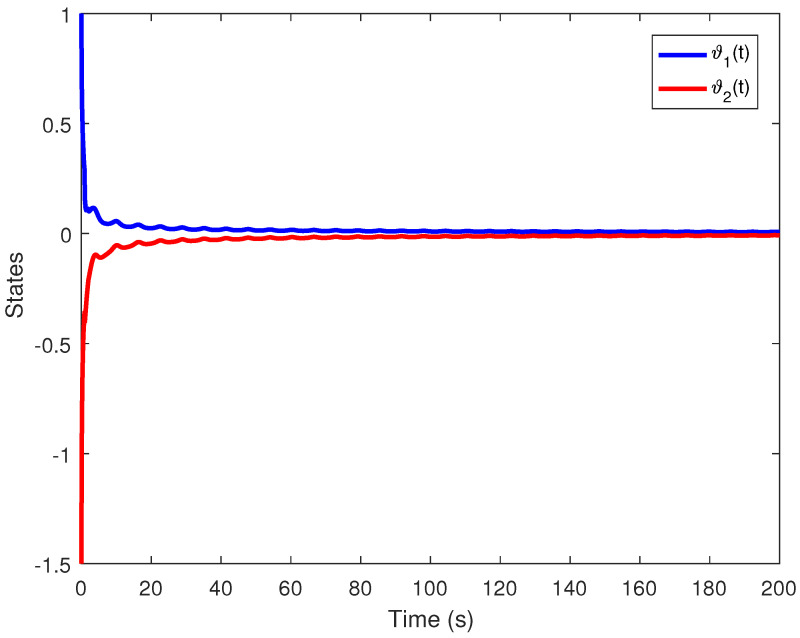
Time evolution for Example 2 with q=0.6.

*Figure 5, Figure 6, Figure 7 and Figure 8 represent the time response of the system with q=0.9,0.8,0.7,0.6. From these figures, we infer that decreasing q yields a larger settling time of the system response. The results also reveal a satisfactory system behavior.*

## 7. Conclusions

The output feedback control strategy of a class of FO neutral-type delay systems was studied in this paper. The stability criteria for the GGC of this type of systems considering a time-varying parametric uncertainty and delayed input were derived via the Lyapunov theory. The output feedback control technique was used, and the system asymptotic stability was achieved. The technique was applied to case studies and its behavior was verified. In future research, the stability of FO neutral-type delay systems with nonlinearity and varying delay will be addressed.

## Figures and Tables

**Table 1:** Comparison in terms of different aspects of FO model.

Related Works	FO Model Properties		Delay Type		
	Nonlinearity	Parametric Uncertainty	Constant	Varying	Neutral
[1]			∗		∗
[2]				∗	
[25]		∗			
[26]		∗			
[43]	∗	∗	∗		
[44]	∗	∗			
[45]		∗	∗		
**Current work**		**∗**	**∗**		**∗**

**Table 2:** Comparison in terms of controller types.

Related Works	Controller Type			
	State-Feedback	Output-Feedback		Optimality
		Static	Dynamic	
[1]	∗			∗
[2]	∗			
[25]			∗	
[26]		∗		
[43]	∗			∗
[44]	∗			∗
[45]	∗			∗
**Current work**			**∗**	**∗**
